# Ion adsorption-induced reversible polarization switching of a van der Waals layered ferroelectric

**DOI:** 10.1038/s41467-021-20945-7

**Published:** 2021-01-28

**Authors:** Dong-Dong Xu, Ru-Ru Ma, Ai-Ping Fu, Zhao Guan, Ni Zhong, Hui Peng, Ping-Hua Xiang, Chun-Gang Duan

**Affiliations:** 1 Key Laboratory of Polar Materials and Devices (MOE) and Department of Electronics, East China Normal University, Shanghai, 200241 China; 2 State Key Laboratory of Bio-Fibers and Eco-Textiles, College of Chemistry and Chemical Engineering, Qingdao University, Qingdao, 266071 China; 3 Collaborative Innovation Center of Extreme Optics, Shanxi University, Taiyuan, Shanxi 030006 China

**Keywords:** Two-dimensional materials, Ferroelectrics and multiferroics

## Abstract

Solid-liquid interface is a key concept of many research fields, enabling numerous physical phenomena and practical applications. For example, electrode-electrolyte interfaces with electric double layers have been widely used in energy storage and regulating physical properties of functional materials. Creating a specific interface allows emergent functionalities and effects. Here, we show the artificial control of ferroelectric-liquid interfacial structures to switch polarization states reversibly in a van der Waals layered ferroelectric CuInP_2_S_6_ (CIPS). We discover that upward and downward polarization states can be induced by spontaneous physical adsorption of dodecylbenzenesulphonate anions and N,N-diethyl-N-methyl-N-(2-methoxyethyl)-ammonium cations, respectively, at the ferroelectric-liquid interface. This distinctive approach circumvents the structural damage of CIPS caused by Cu-ion conductivity during electrical switching process. Moreover, the polarized state features super-long retention time (>1 year). The interplay between ferroelectric dipoles and adsorbed organic ions has been studied systematically by comparative experiments and first-principles calculations. Such ion adsorption-induced reversible polarization switching in a van der Waals ferroelectric enriches the functionalities of solid-liquid interfaces, offering opportunities for liquid-controlled two-dimensional ferroelectric-based devices.

## Abstract (web summary)

Whether it is possible to achieve polarization inversion in a ferroelectric without any energy consumption is an open question. Here, the authors demonstrate an energy-free approach to control the polarization state of CuInP_2_S_6_, a typical room-temperature van der Waals layered ferroelectric.

## Introduction

Ferroelectric materials are known for their spontaneous polarization that can be reversibly switched by an electric field. The ability to control the polarization states lies at the heart of ferroelectric physics and forms the basis of ferroelectric applications^1,2^, such as polarization-controlled electronic^3^, magnetic^4^, optical^5^, mechanical^6^, and other functional properties^7^. On the other hand, how to control the ferroelectric polarization states has become a topic of general concern, which can determine the application scope of ferroelectrics at the fundamental level. Although applying an external electric field is the most common approach to switching polarization state, a growing body of research indicates that voltage-induced electrochemical phenomena can be remarkable ranging from vacancy ordering, deposition to surface damage^8–11^. Preeminent examples include well-known ferroelectric ion conductor KTiOPO_4_^12,13^ and its homologous compounds^14,15^ of which the periodical polarization switching utilizing electric field suffers from their high conductivity^16–18^.

Recently CuInP_2_S_6_ (CIPS), a typical room-temperature van der Waals (vdW) layered ferroelectric crystal^19–21^, has attracted intensive attention due to its unexpected features including giant negative piezoelectricity^22^, tunable quadruple-well ferroelectric nature^23^, negative capacitance characteristic^24^, and the interplay between ferroelectricity and ionic conductivity^25–27^. These unique characteristics not only bring insights into the fundamental research^28,29^ but also provide opportunities for diverse applications of layered ferroelectrics^30,31^. However, when electrically switching ferroelectric domains, the polar structure and ferroelectricity of CIPS can be readily damaged^32,33^ due to its high ionic conductivity^34,35^. Because the ionic conductivity and polarization switching have common atomic origin-Cu atoms, it seems difficult to achieve a balance between these two processes. Therefore, beyond the electrical switching, a simple and effective method of controlling polarization and simultaneously suppressing ionic conductivity is in urgent demand, which may help in expanding the application scope of layered ferroelectrics. It has been reported that various external stimuli (e.g., temperature^36,37^, mechanical stress^38,39^, chemical reactions^40,41^, and light^42,43^) can be used for the polarization control of conventional perovskite-type oxide ferroelectrics. However, the interactions between ferroelectric dipoles and external stimuli have been rarely investigated in the family of vdW layered ferroelectric materials^44^, although there are stark differences in terms of both atomic packing and bonding energies.

Here we report the interplay between ferroelectric dipoles and adsorbed ions at CIPS–liquid interfaces. In general, interfacial structures (absorbed species and their depth profiles) strongly depend on the direction of ferroelectric polarization^45^. The intrinsic surface charges of ferroelectrics are screened by adsorbed counter ions/molecules to reduce the energy of the depolarizing field^46^. In this paper, we show the converse effect—the adsorbed ions at ferroelectric interfaces can control the polarization states. Large-area reversible polarization switching of CIPS without fossil energy consumption and structural damage has been experimentally achieved via altering the type of adsorbed ions at room temperature. The observed phenomena could deepen the understanding of complex interfacial interactions and may provide a paradigm for artificially manipulating ferroelectric–liquid interfacial structures leading to unexplored interfacial effects.

## Results

### Ionic-liquid-induced large-area polarization switching

**Fig. 1:**
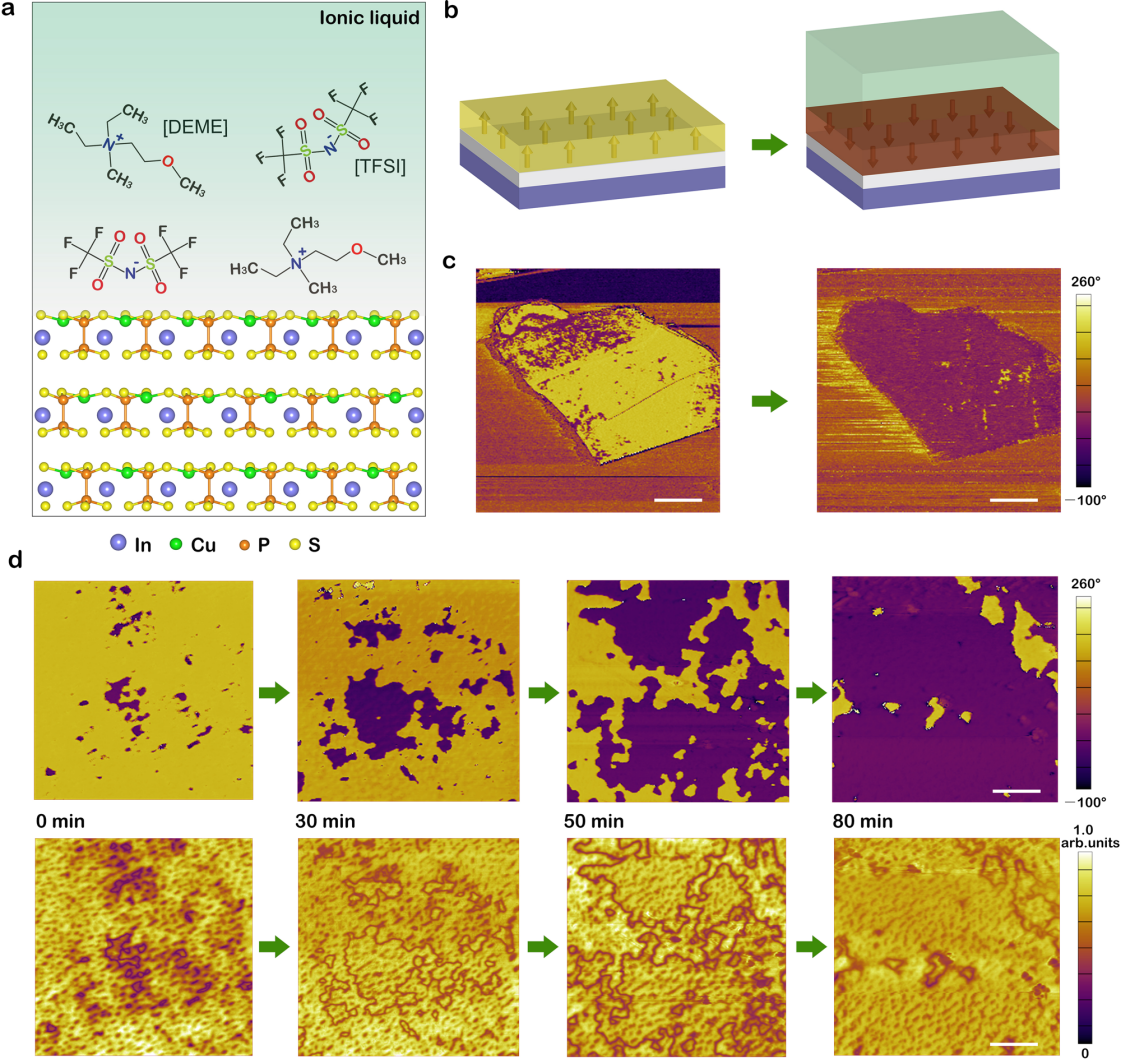
Ferroelectric domain switching of vdW layered CIPS induced by the ionic liquid. **a** Sketch of the layered ferroelectric–ionic liquid interface, highlighting the atomic-level structure of CIPS and the molecular structure of ionic liquid [DEME][TFSI]. **b, c** Schematic diagrams (**b**) and corresponding PFM phase images (**c**) of exfoliated CIPS flakes on an ultra-flat Pt/SiO_2_ substrate, showing polarization switching from upward (yellow) to downward (purple) polarization state induced by ionic liquid [DEME][TFSI]. The arrows represent ferroelectric dipoles of CIPS. Scale bar in (**c**), 1.5 μm. **d** Time evolution of PFM phase (top panel) and corresponding amplitude signals (bottom panel) for a 94 nm-thick flake. Scale bar in (**d**), 1 μm.

CIPS is an excellent model system to study the diverse coupling effects between ferroelectric dipoles and external stimuli due to its room temperature ferroelectricity (Curie temperature *T*_c_ ~ 315 K). Below *T*_c_, the ferroelectric phase is generated by the antiparallel displacement of Cu and In sublattices along with a symmetry reduction from non-polar *C*2/*c* to polar *Cc* space group^47^. As shown in Fig. 1a, the off-centered ordering of Cu sublattice corresponds to upward polarization. The room-temperature ferroelectricity of CIPS samples used in this work has been examined in our previous studies^25^. For this study, about 84 nm thick CIPS nanoflakes were obtained by mechanical exfoliation onto an ultra-flat Pt/SiO_2_ substrate. The initial polarization states feature both upward (yellow domains) and downward polarization (purple domains) that were characterized by piezoresponse force microscopy (PFM). After exposure to N,N-diethyl-N-methyl-N-(2-methoxyethyl)-ammonium bis-(trifluoromethanesulfonyl)imide ([DEME][TFSI]), a kind of room-temperature ionic liquid composed of large organic cations [DEME] and anions [TFSI] (Fig. 1a), the initial upward polarization states were fully switched to downward polarization (Fig. 1b, c). In contrast, the original downward polarization states remained unchanged. It has been reported that large crystallites can be formed at the surface of CIPS when electrically switching ferroelectric domains^32,33^. In this work, we noted that no external electric field was applied and no surface deformations were observed (see Supplementary Fig. 1). Experimental details can be found in Supplementary Note 1 and Supplementary Fig. 2. It is worth noting that this interesting phenomenon is highly reproducible for CIPS flakes with varying thicknesses (see Supplementary Figs. 1, 3, and 4).

To further study the dynamics of polarization switching induced by the ionic liquid, local domain structures changing over time has been carefully investigated. Figure 1d shows the time evolution of the PFM phase (top panel) and amplitude signals (bottom panel) of CIPS nanoflake with a thickness of 94 nm. By increasing the duration of exposure to ionic liquid [DEME][TFSI], the downward ferroelectric domains expanded dramatically, which is clearly verified by the corresponding evolution of amplitude images. The switched area as a function of time for samples with various thicknesses is summarized in Supplementary Fig. 5. The switching rate not obviously depends on the thickness of prepared samples ranging from ~30 nm to ~100 nm.

### Interfacial effect at CIPS–ionic liquid interfaces

**Fig. 2:**
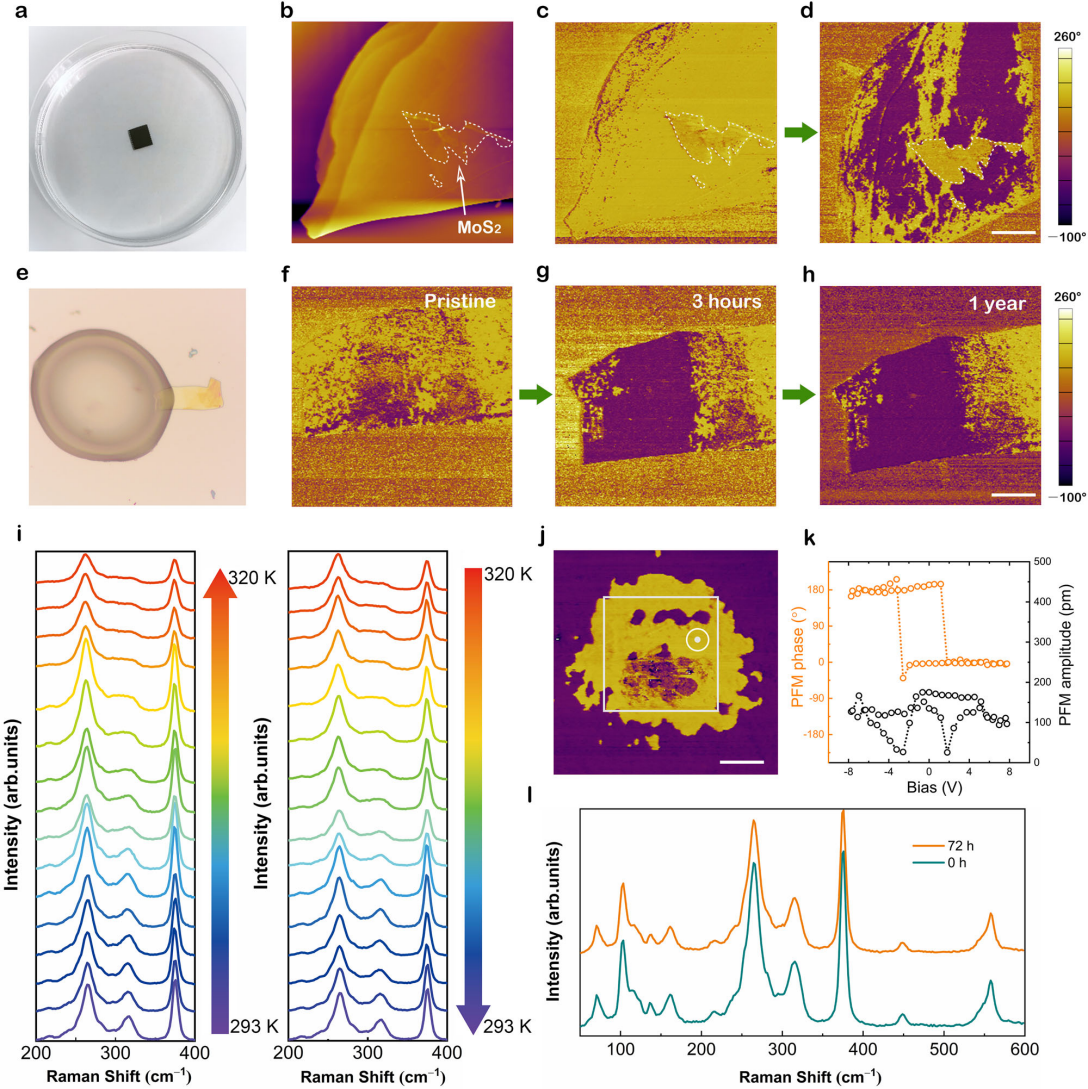
Interfacial effect-induced polarization switching of layered CIPS. **a** Photograph of the ionic liquid treatment process. **b** The topography of a MoS_2_ (4 nm)/CIPS (87 nm) heterojunction. **c**, **d** The PFM phase images before (**c**) and after (**d**) ionic liquid treatment. Scale bar in (**d**), 5 μm. **e** Photograph of a CIPS nanoflake partially covered by ionic liquid [DEME][TFSI]. **f**–**h** PFM phase images of the nanoflake in (**e**) after exposing to ionic liquid for 0 h (**f**), 3 h (**g**), and 1 year (**h**), respectively. Scale bar in (**h**), 5 μm. **i**–**l** Characterization of the ferroelectricity in CIPS samples treated by ionic liquid for 72 h, including variable-temperature Raman spectra (**i**) showing a reversible ferroelectric phase transition, switchable phase pattern (**j**) indicating that downward polarized domains can be switched back again by applying a positive voltage, 180° phase switching and well-defined butterfly loop (**k**) and the comparison of Raman spectra before and after ionic liquid treatment (**l**). Scale bar in (**j**), 500 nm.

For layered transition-metal chalcogenophosphates, another attractive feature is the large vdW gap offering sufficient space for ion intercalation^48^. It has been reported that the intercalation of organic molecules plays a significant role in controlling structurally, electronically, and magnetically ordered states^49^. To examine the possibility of intercalation of [DEME] or [TFSI] ions, the thickness and morphology of CIPS flakes before and after ionic liquid treatment have been carefully checked. The flat surface and unchanged thickness evidently excluded the ion intercalation mechanism (see Supplementary Figs. 1, 3, and 4) because ion intercalation can lead to surface wrinkle and bulk swelling of layered materials. To prove this point, we carried out a contrast experiment. Figure 2a shows the photograph of the ionic liquid treatment process. Before exposure to the ionic liquid, a 4 nm thick MoS_2_ nanoflake was placed on the surface of CIPS to prevent the ionic liquid from contacting with CIPS (Fig. 2b). Note that we can easily detect the response signals of ferroelectric domains beneath such ultra-thin MoS_2_ film (see Supplementary Fig. 6). It can be observed clearly that the region covered by MoS_2_ owns an identical upward polarization state with surrounding areas (Fig. 2c). After ionic liquid treatment, large-area polarization switching was induced by the ionic liquid, except the coverage domains giving a sharply defined identical domain shape with that of MoS_2_ (Fig. 2d). These results reveal an interfacial effect evidently rather than a bulk effect with respect to ion intercalation.

Figure 2e shows a strip-shaped CIPS nanosheet partially covered by the ionic liquid. Only the coverage area experienced a polarization switching process yet again proving the role of solid–liquid interface (Fig. 2f, g). More surprisingly, after removing the ionic liquid, the switched domain pattern displays almost no change after 1 year (Fig. 2h), suggesting robust stability. The super-long retention time is comparable to the longest retention time of classical perovskite-type oxide ferroelectrics electrically poled by a scanning probe^50,51^.

In addition to durability, structure stability is a very important aspect in practical applications, especially for 2D layered ferroelectric materials. As shown in Fig. 2i, variable-temperature Raman spectra have been performed on the CIPS samples treated by ionic liquid for 72 h. With the temperature increasing, the sharp peak at 313 cm^−1^ gradually disappears indicating a typical ferroelectric-paraelectric transition and vice versa^25^. The clear phase contrast within the white box (Fig. 2j) written by a PFM tip verifies the well-preserved switchable dipoles in CIPS. The distinct 180° phase switching and well-defined butterfly loop in Fig. 2k also prove this point. Moreover, the Raman spectra make no difference before and after ionic liquid treatment (Fig. 2l). These results not only demonstrate that the polar crystal structure and switchable ferroelectricity of CIPS have not been damaged by the ionic liquid but also help us rule out the case of ion intercalation reaction in terms of unchanged structures. Therefore, we attribute the domain switching mechanism to a solid–liquid interface effect. Since ionic liquids are entirely composed of large ions, the physical adsorption of ions at the CIPS surface may account for the above polarization switch phenomenon.

### Mechanism discussion and reversible switching

In the material systems of perovskite-type oxide ferroelectrics, it has been reported that H^+^ adsorption and chemical bonding across ferroelectric–water interface can induce polarization switch from upward to downward state^40^. In that case, the formation of chemical bonds (metal–O–H at the ferroelectric surface) rather than physical adsorption of molecules gives rise to a significant displacement of B-site cations. While it is hard to form chemical bonding at the surface of vdW layered ferroelectrics at room temperature due to the absence of dangling bonds. To test whether H^+^ plays a role in the polarization switching process, CIPS nanoflakes were immersed in an acidic aqueous solution with pH = 3. As a result, after 4 h, both surface morphology and polarization states did not change ruling out the possible effect of H^+^ and H_2_O experimentally (see Supplementary Fig. 7). The good surface morphology, distinguishable domain structures, and constant thickness clearly suggest the high-quality polar structures of CIPS flakes before and after acidic solution treatment.

**Fig. 3:**
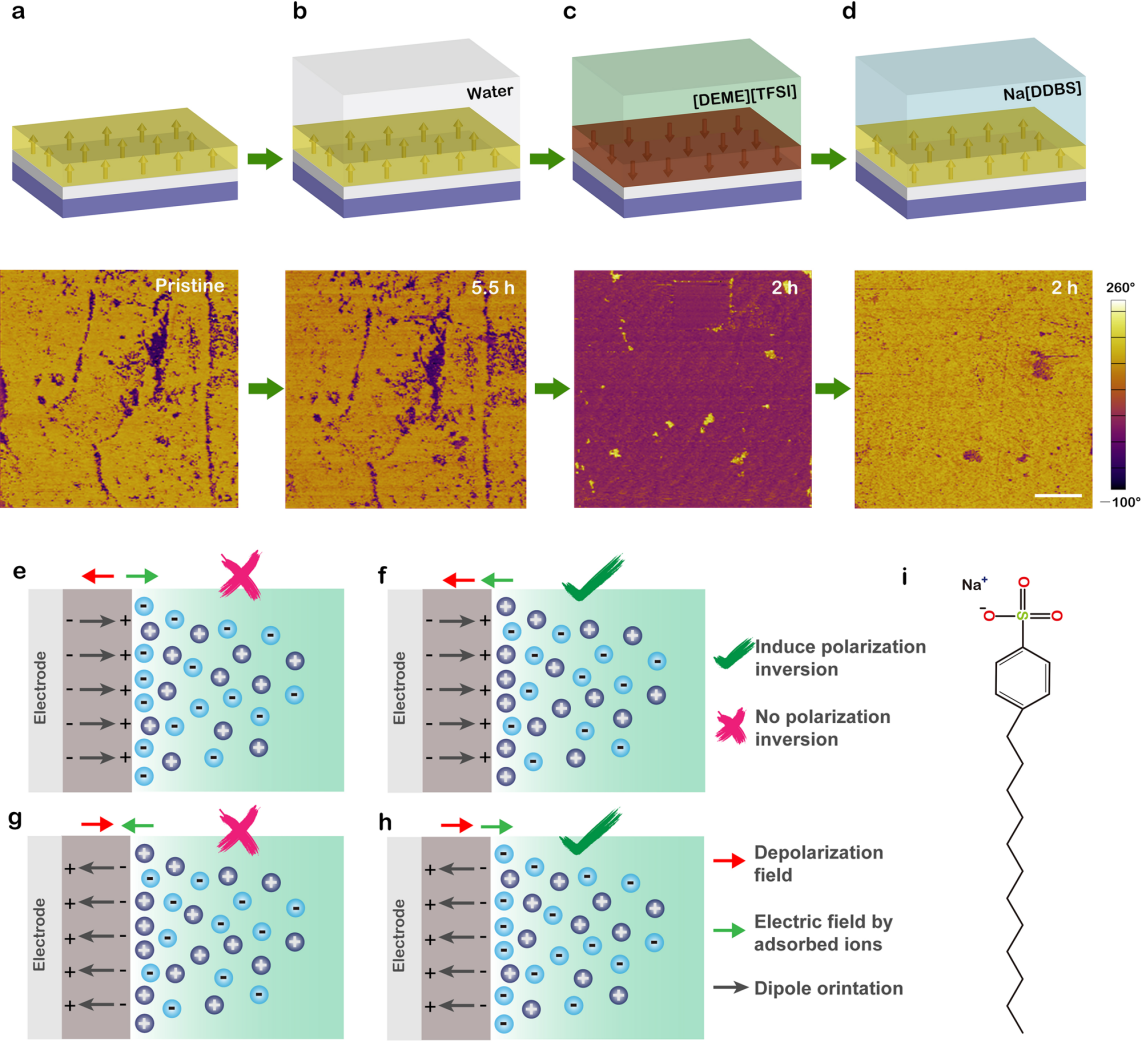
Ion adsorption-induced reversible polarization switching. **a**–**d** Schematic diagrams and corresponding PFM phase changes of the same CIPS nanoflake after exposing to water (**b**), ionic liquid [DEME][TFSI] (**c**) and Na[DDBS] solution (**d**), respectively. Scale bar in (**d**), 3 μm. **e**–**h** Diagrams of four possible ion adsorption scenarios for upward (**e**), (**f**), and downward (**g**), (**h**) polarization surfaces, respectively. **i** The molecular structure of Na[DDBS] surfactant.

In order to make it clear which kind of ions interact with ferroelectric dipoles at the solid–liquid interface, consecutive experiments have been performed on the same CIPS sample as shown in Fig. 3a–d. We note that a small amount of water is inevitable in the as-received ionic liquid (typically in the order of ppm). To obtain further insights into the possible effect of H_2_O, deionized water was used to cover the CIPS surface directly. After 5.5 h of exposure to water, no obvious domain switching phenomenon has been observed indicating that water molecules cannot efficiently affect the polarization states yet again (Fig. 3a, b). In contrast, ionic liquid [DEME][TFSI] can switch the polarization states remarkably only in 0.5 h (see Supplementary Fig. 8) and realize a full switching after 2 h (Fig. 3c). It should be noted that to eliminate the impact of residual moisture content, the CIPS flakes covered by ionic liquid were placed in a vacuum and the ionic liquid used in this work has been heated at 423 K for 2 h in a vacuum environment. In addition, the influence of acetone and ethanol treatment used to remove residual ionic liquid on domain structures could be neglected (see Supplementary Fig. 9). These results confirm that only the organic cations/anions inside ionic liquid play an integral role at ferroelectric interfaces.

Now the question turns to be which kind of ions inside ionic liquid plays a role. Given that mainly anions [TFSI] adsorb on the positively charged surface (upward polarization), the anions [TFSI] can screen the intrinsic surface charge of CIPS and offer an opposite electric field to the depolarization field (Fig. 3e). For clarity, all kinds of cations/anions are represented by positively/negatively charged pellets, respectively. The energy of this depolarization field can be reduced by the negative charges at CIPS–ionic liquid interfaces. Actually, this case has been reported for polarized ferroelectrics in ionic liquids/aqueous solutions^45,52^. Charged ferroelectric surfaces can control the formation of the electric double layer (EDL) via long-range electrostatic interaction. A typical compact layer (Helmholtz layer) is formed by counter ions at first, to which diffusion layer is developed adjacently. However, if this scenario holds true, the initial upward polarization state should be more stable and the polarization inversion process will not happen, which is in contradiction with our experimental observations. Therefore, it is natural to take cations [DEME] into consideration. Assuming that mainly cations [DEME] adsorb on the positively charged surface with an upward polarization state (Fig. 3f), the adsorbed like-charges provide an additional electric field with the same direction of depolarization field driving a significant displacement of Cu atom in layers of CIPS. Consequently, the polarization direction could be changed, which is well consistent with our experiment phenomena although the attraction behavior between positively charged CIPS surfaces and [DEME] cations is apparently counterintuitive. In a broader perspective, the attraction phenomena between like-charged objects have been extensively studied in many systems such as macroions ranging from colloids, polymers to DNA solutions^53^. In the case of ionic liquids, both molecular dynamic simulations^54^ and experimental evidences^55^ have revealed a cation-rich interface between ionic liquid and positively charged surface because of the specific adsorption of cations in ionic liquid. These reports give us a hint that the EDL structures would be determined not only by long-range electrostatic forces but also by some short-range interactions. Moreover, it is well-known that ionic liquids with surface-active cations often serve as cationic surfactants due to their good surface adsorbability^56,57^. It is reasonable to infer that the used cations [DEME] and its analogs may exhibit a strong adsorption effect to the CIPS surface. To emphasize the general nature of the adsorption effect of cations, we performed similar experiments utilizing ionic liquids [DEME][BF_4_] with new anions [BF_4_] and [EMIM][BF_4_] containing new cations [EMIM]. The domain switching phenomena resembling the experimental observations in [DEME][TFSI] are displayed in Supplementary Figs. 10 and 11. These results demonstrate the universality of ionic liquid-induced polarization switching in CIPS.

Based on the above discussions, we attribute the ionic liquid-induced polarization switching to the adsorption of cations at the CIPS–ionic liquid interface. However, the lack of in situ microscopic techniques makes it difficult to trace ferroelectric–liquid interfacial structures. To understand this mechanism, we conducted a simple experiment circumventing technical difficulties. If the adsorption of large quaternary ammonium cations [DEME] can induce the switching of upward polarization state, the downward polarized domains can be further switched by the surface adsorption of large anions in the same way (Fig. 3h). In other words, we can artificially construct desirable solid–liquid interfacial states just by selecting the appropriate adsorbed ions to switch the ferroelectric polarization states reversibly. Sodium dodecylbenzenesulphonate (Na[DDBS]), a famous anionic surfactant (the main ingredient of detergent), was chosen to provide suitable large anions [DDBS]. The [DDBS] anion has a long alkyl chain exhibiting strong adsorption capacity for solids^58^. Its molecular structures can be found in Fig. 3i. As expected, downward polarized domains were reversed back after immersed into aqueous solutions of Na[DDBS] (Fig. 3d). The direction of back-switched polarization was verified by applying an external electric field (see Supplementary Fig. 8r). We also carried out repeated experiments on other CIPS flakes and observed analogous phenomena (see Supplementary Fig. 12). Similarly, these results cannot be explained by the scenario of cation (Na^+^) adsorption as shown in Fig. 3g. Raman spectra suggest that the polar structures of CIPS are well-preserved after Na[DDBS] treatment (see Supplementary Fig. 13).

### Simulation of the adsorption behaviors at CIPS–liquid interfaces

**Fig. 4:**
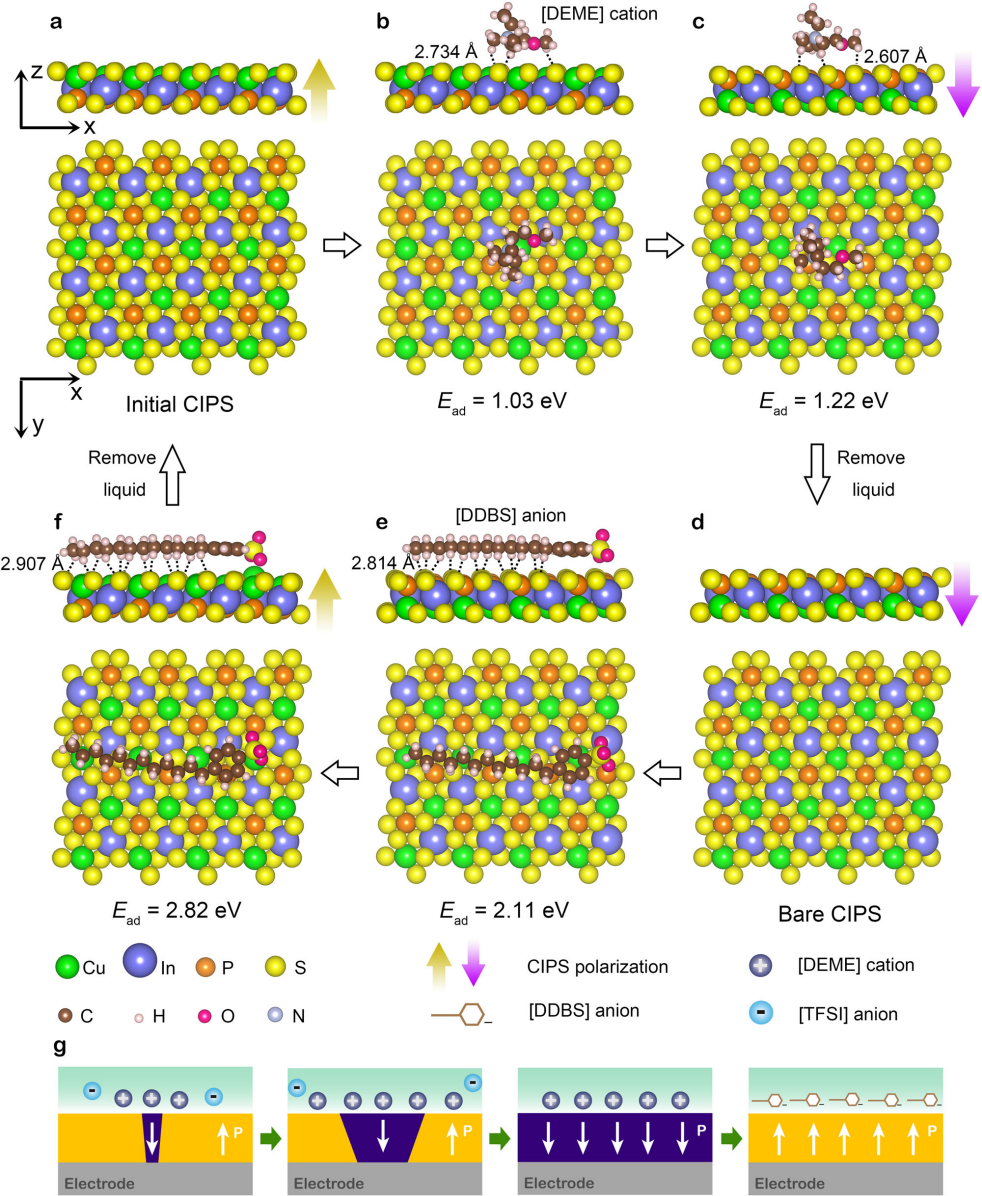
Modeling of the adsorption geometry and interactions between [DEME]/[DDBS] ions and CIPS surfaces. **a** Initial CIPS with an upward polarization state. **b** Adsorption of [DEME] cations from ionic liquid [DEME][TFSI] onto the CIPS surface. **c** Ferroelectric switching induced by the adsorption of [DEME] cations, corresponding to a significant displacement of Cu atoms. **d** After removing ionic liquid, bare CIPS samples with opposite dipole orientations are obtained. **e** Adsorption of [DDBS] anions from surfactant solution onto the CIPS surface of (**d**). **f** Back-switch to the initial upward polarization state. The dotted lines represent C–H···S bonds where only the shortest bond length is labeled. **g** Schematic diagram of the dynamic polarization switching process.

In order to obtain molecular insights into the adsorption behaviors of [DEME] cations and [DDBS] anions on CIPS, first-principles calculations have been performed using the DMol^3^ code^59,60^ (Fig. 4). The generalized gradient approximation with Perdew–Burke–Ernzerhof (PBE) functional and the DFT semi-core pseudo-potentials with double numerical atomic basis set plus polarization were employed^61^. To compensate for the poor description of the weak vdW interactions by the popular PBE functional, an empirical dispersion-corrected density functional theory (DFT-D) approach proposed by Grimme was used^62^. All the calculations are based on the 24.4 Å × 21.2 Å × 30 Å supercells to accommodate the large-size [DEME] and [DDBS] ions. The adsorption energy can be expressed as:

1 $$E_{{\mathrm{ad}}} = E_{{\mathrm{surface}}} + E_{{\mathrm{ion}}}-E_{{\mathrm{surface}}{\hbox{-}}{\mathrm{ion}}}$$

where *E*_surface_, *E*_ion_, and *E*_surface-ion_ is the total energy of bare CIPS surface, an isolated ion, and the optimized CIPS with adsorbed species, respectively. Adsorption energies of cations and anions onto the same surface are shown in Supplementary Figs. 14 and 15. [DEME] cations and [DDBS] anions are preferred to be adsorbed by CIPS surface with initial upward and downward polarizations, respectively (Supplementary Note 2). During the ion adsorption, the adsorption energy differences between positive and negative surfaces make polarization switching energetically favorable leading to a significant displacement of Cu atom with respect to the P_2_S_6_-defined framework. The driving force is the reduction in the total energy of [DEME]-CIPS and [DDBS]-CIPS systems. The switching mechanism is summarized in Fig. 4. One might question that, before polarization switching, there is always coulomb repulsion between adsorbed [DEME] cations (DDBS anions) and positive (negative) surface charges of CIPS with an initial upward (downward) polarization state to prevent adsorption. Actually, besides vdW forces between ions and surfaces, the sulphur-mediated C–H···S bonds (dotted lines in Fig. 4) can provide attractive forces to overcome the Coulomb repulsion effect. The relatively strong vdW forces and hydrogen bonding compared to electrostatic interaction can be confirmed by the comparison of adsorption energies of large [DEME] cation and small Na^+^ ion at CIPS surfaces quantitatively (see Supplementary Fig. 16 and Supplementary Note 3). It should note that the strength of C–H···S bonds lies between strong chemical bonds (e.g., covalent/ionic bonds) and vdW forces^63^. Our first-principles calculations indicate that the bond lengths of C–H···S bonds vary from 2.607 to 3.696 Å. On the other hand, compensating charges should be on the CIPS surface (see Supplementary Fig. 17) according to the generally accepted surface screening mechanism. Actually, partial screening of polarization charges is favorable for the adsorption of [DEME] cations ([DDBS] anions) and suppress the adsorption of [TFSI] anions (Na^+^ ions) (see Supplementary Note 2). These results pave a way to switch the ferroelectric polarization reversibly. Especially, it demonstrates a potential advantage in ferroelectric systems that have leakage problems, e.g., ferroelectric metal^64–66^, where the conventional method is not applicable.

To summarize, we demonstrate an ion adsorption-induced polarization switching of a vdW layered ferroelectric CIPS. Analogous to applying voltage, the adsorbed ionic charges can introduce an electric field to switch ferroelectric domains reversibly depending on the sign of ionic charges adsorbed by ferroelectric surfaces. Large-area polarization switching of CIPS has been achieved without an external electric field at room temperature, circumventing the polar structure damage caused by high ionic conductivity. The ability to manipulate domain orientations holds promise as a method to realize energy-free erasable-type printing by a mask (Fig. 2a–h).

Compared with dipole orientation-dependent ionic/molecular adsorption, the converse effect—ionic control of dipole orientation provides a more facile and visual approach to probe the complex interactions at ferroelectric surfaces. Ferroelectric response to the surrounding chemical environment gives us an opportunity to clarify the specific role and relative strength among these interactions determining ionic adsorption and charge transfer process across the solid–liquid interface. Therefore, vdW layered ferroelectric can be an excellent model system for studying the interplay between surface dipoles and external chemical stimuli. Moreover, these observations highlight the role of the chemical environment that can be used in exploring polarization-dependent applications including chemical sensors, data storage, catalysis, etc.

## Methods

### Sample preparation

High-quality CIPS single crystals were purchased from HQ Graphene company. We used ultra-flat Pt(100 nm)/Ti(10 nm)/SiO_2_(300 nm)/Si substrates for CIPS exfoliation. The samples were soaked in ionic liquids or surfactant solutions at room temperature for hours, and then washed by acetone, ethanol, and water for a few seconds, respectively, finally blown by dry nitrogen gas to remove residual liquid. Before the experiment, the ionic liquid used in this work has been heated at 423 K for 2 h in a vacuum environment to eliminate residual moisture content, and then the CIPS flakes covered by ionic liquid were placed in a vacuum. The concentration of Na[DDBS] aqueous solution is 0.5 mol/L.

### PFM measurements

The ferroelectric domain structures and local piezoresponse hysteresis loops were characterized by DART PFM (Asylum Cypher) with conductive Pt-coated silicon cantilevers. A soft tip with a spring constant of 2.8 N m^−1^ was driven with an ac voltage (*V*_ac_ = 0.5 V) under the tip-sample contact resonant frequency (≈320 kHz). For domain switching, a dc voltage was applied to a PFM probe using Litho PFM while the bottom electrode was grounded. Surface potential mapping was imaged using KPFM with nap height 20–30 nm.

### Raman spectroscopy measurements

Raman spectrum was conducted on a Renishaw inVia confocal Raman microscope with a ×100 objective lens (NA = 0.85). The excitation wavelength is 532 nm with spectral resolution narrower than 0.5 cm^−1^ and spatial resolution below 0.5 μm.

### Reporting summary

Further information on research design is available in the Nature Research Reporting Summary linked to this article.

## Supplementary information

Supplementary Information

Reporting Summary

## Data Availability

The data that support the findings of this study are available from the corresponding author upon reasonable request.
