# Supplementary material for: Ion adsorption-induced reversible polarization switching of a van der Waals layered ferroelectric
Source: Nat Commun. 2021 Jan 28;12:655. doi: 10.1038/s41467-021-20945-7 (PMC7844287; doi:10.1038/s41467-021-20945-7)
Supplement: Supplementary file 1 — Supplementary Information [file 41467_2021_20945_MOESM1_ESM.pdf]

# Supplementary Information

# Ion adsorption-induced reversible polarization switching of a van der Waals ferroelectric

Dong-Dong Xu[1,†], Ru-Ru Ma[1,†], Ai-Ping Fu[2], Zhao Guan[1], Ni Zhong[1,3*], Hui Peng[1], Ping-Hua Xiang[1,3*] & Chun-Gang Duan[1,3]

[1] Key Laboratory of Polar Materials and Devices (MOE) and department of electronics, East China Normal University, Shanghai, 200241, China

[2] State Key Laboratory of Bio-Fibers and Eco-Textiles, College of Chemistry and Chemical Engineering, Qingdao University, Qingdao 266071, China

[3] Collaborative Innovation Center of Extreme Optics, Shanxi University, Taiyuan, Shanxi 030006, China

[†]These authors contributed equally to this work.

[*]Correspondence and requests for materials should be addressed to N.Z. (email: nzhong@ee.ecnu.edu.cn) or P.-H.X. (email: phxiang@ee.ecnu.edu.cn).

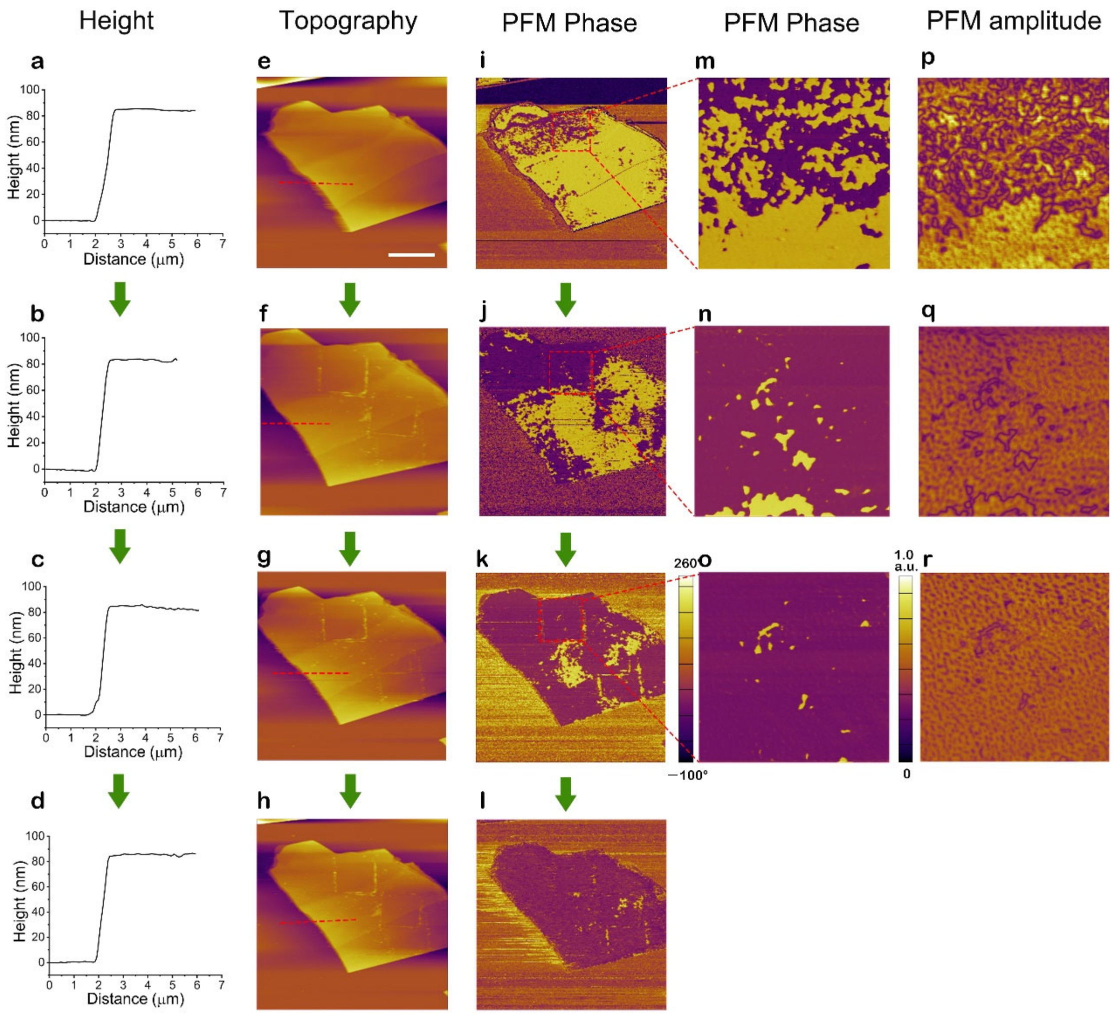


**Supplementary Figure 1. Evolution of ferroelectric domains in CIPS. a-i** Time evolution of the height (**a-d**), topography (**e-h**) and PFM phase images (**i-l**) of a 84-nm thick CIPS flake after exposure to ionic liquid [DEME][TFSI]. Scale bar in (**e**) is 1.5 μm. **m-r** The enlarged local PFM phase (**m-o**) and amplitude signals (**p-r**).

**Supplementary Note 1. Experimental details**

1. The direction of polarization. To distinguish the direction of polarization before and after treatment, the local electrical switching of CIPS has been performed for each PFM measurement as shown in Supplementary Figs. 2 and 8. Such method also confirms the well-preserved ferroelectricity after ionic liquid treatment.
2. The height and topography. Except minor scraping by PFM tips, there are no changes of the height and topography before and after treatment.

3. Ionic liquid. Ionic liquids usually exhibit extremely low vapor pressures (typically ≈$10^{-10}$ Pa at 25 °C) and high thermostability. Therefore, residual moisture content can be eliminated via heating in vacuum.

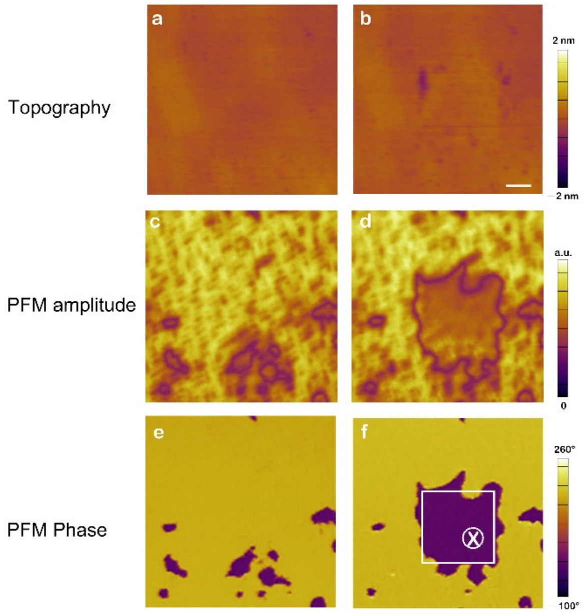


**Supplementary Figure 2. Electrical switching of ferroelectric domains to identify the direction of polarization.** The topography (**a**, **b**), PFM amplitude signals (**c, d**) and corresponding phase images (**e, f**) of a CIPS flake before and after applying a positive voltage. Scale bar in (**d**) is 1 μm. The purple region corresponds to the downward polarization state.

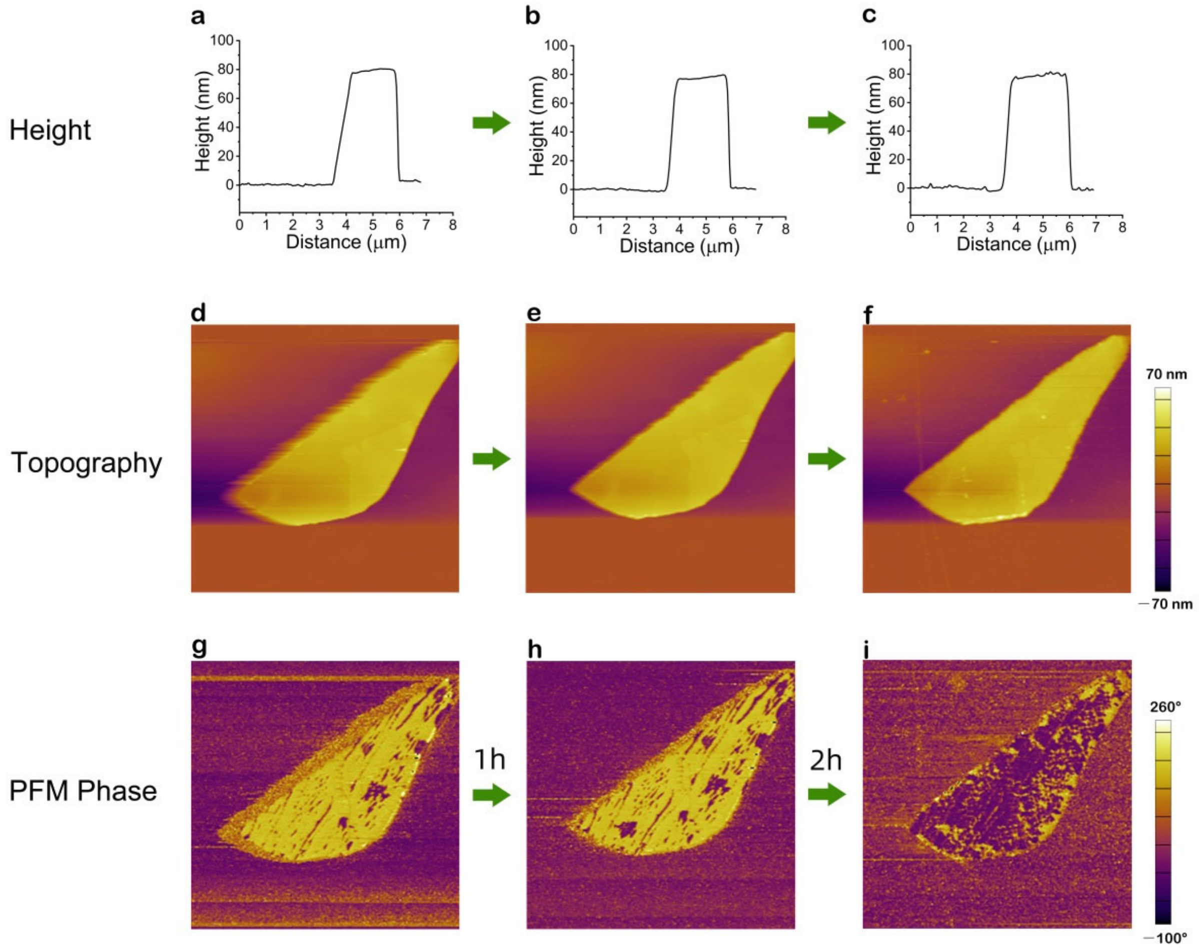


**Supplementary Figure 3. Ionic liquid [DEME][TFSI]-induced large-area polarization switching of CIPS.** Time evolution of the height (**a-c**), topography (**d-f**) and corresponding phase (**g-i**) of an 80-nm thick CIPS flake after exposure to ionic liquid [DEME][TFSI].

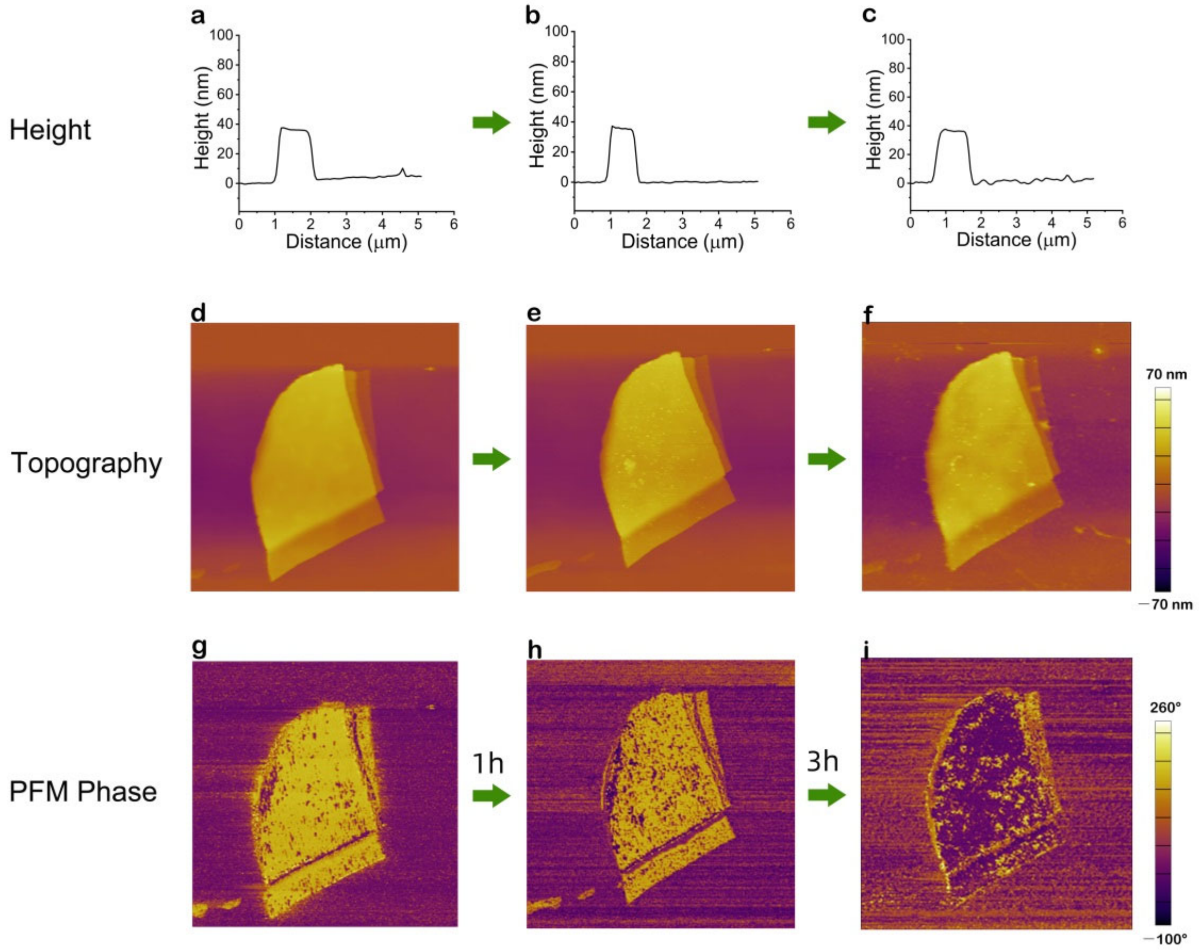


**Supplementary Figure 4. Ionic liquid [DEME][TFSI]-induced large-area polarization switching of CIPS.** Time evolution of the height (**a-c**), topography (**d-f**) and corresponding phase (**g-i**) of a 37-nm thick CIPS flake after exposure to ionic liquid [DEME][TFSI].

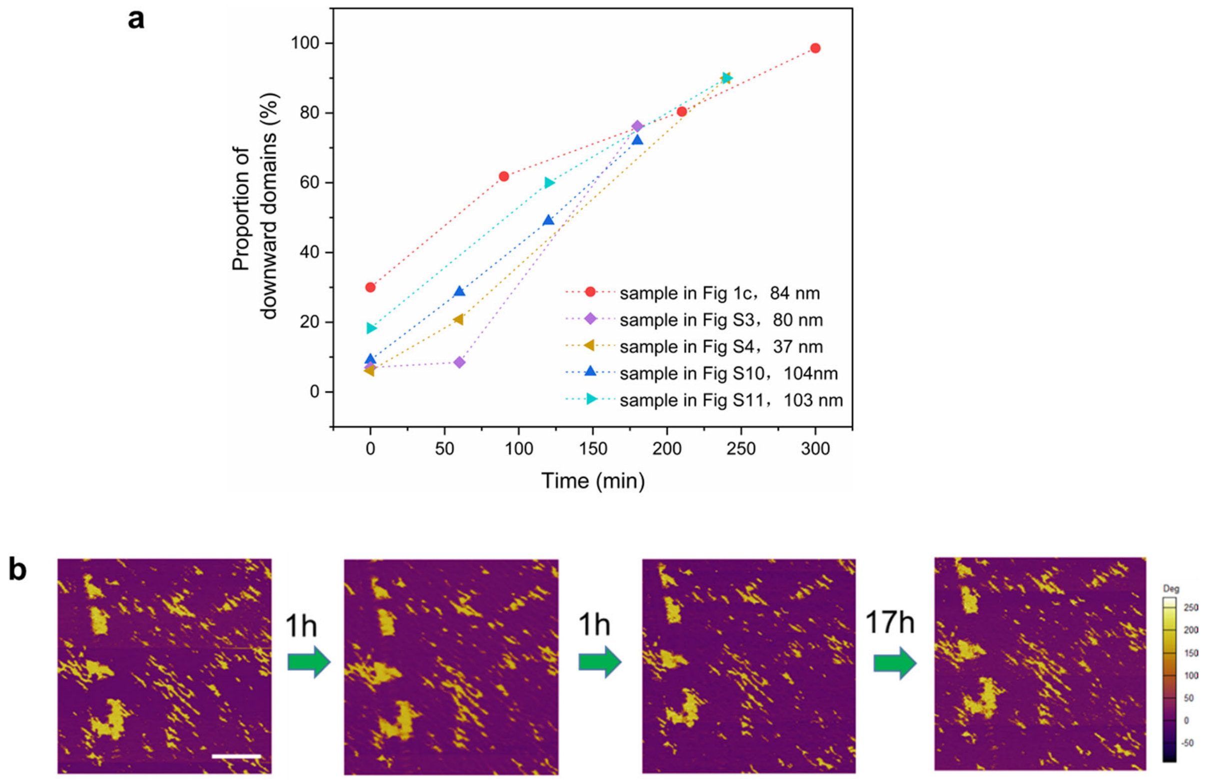


**Supplementary Figure 5. Time evolution of ferroelectric domain structures in CIPS. a** The area of downward domains as a function of time for samples with various thickness ranging from ~30 nm to ~100 nm. **b** Time evolution of PFM phases in a CIPS bulk sample with thickness of ~ 0.6 μm. These results indicate that the polarization of the thicker bulk CIPS cannot be switched by ionic liquid. Scale bar is 2 μm.

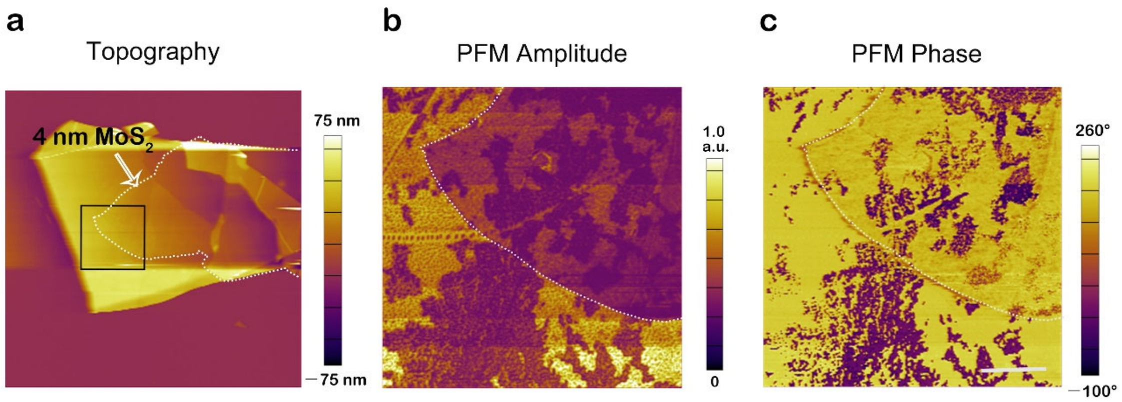


**Supplementary Figure 6. The domain structures of a $MoS_2$/CIPS heterojunction. a** A 4 nm thick $MoS_2$ nanoflake was placed on CIPS. **b, c** The corresponding PFM amplitude signals (**b**) and phase images (**c**) of the region marked in (**a**). These results indicate that ferroelectric domains beneath $MoS_2$ thin film can be detected by PFM. Scale bar in (**c**) is 2 μm.

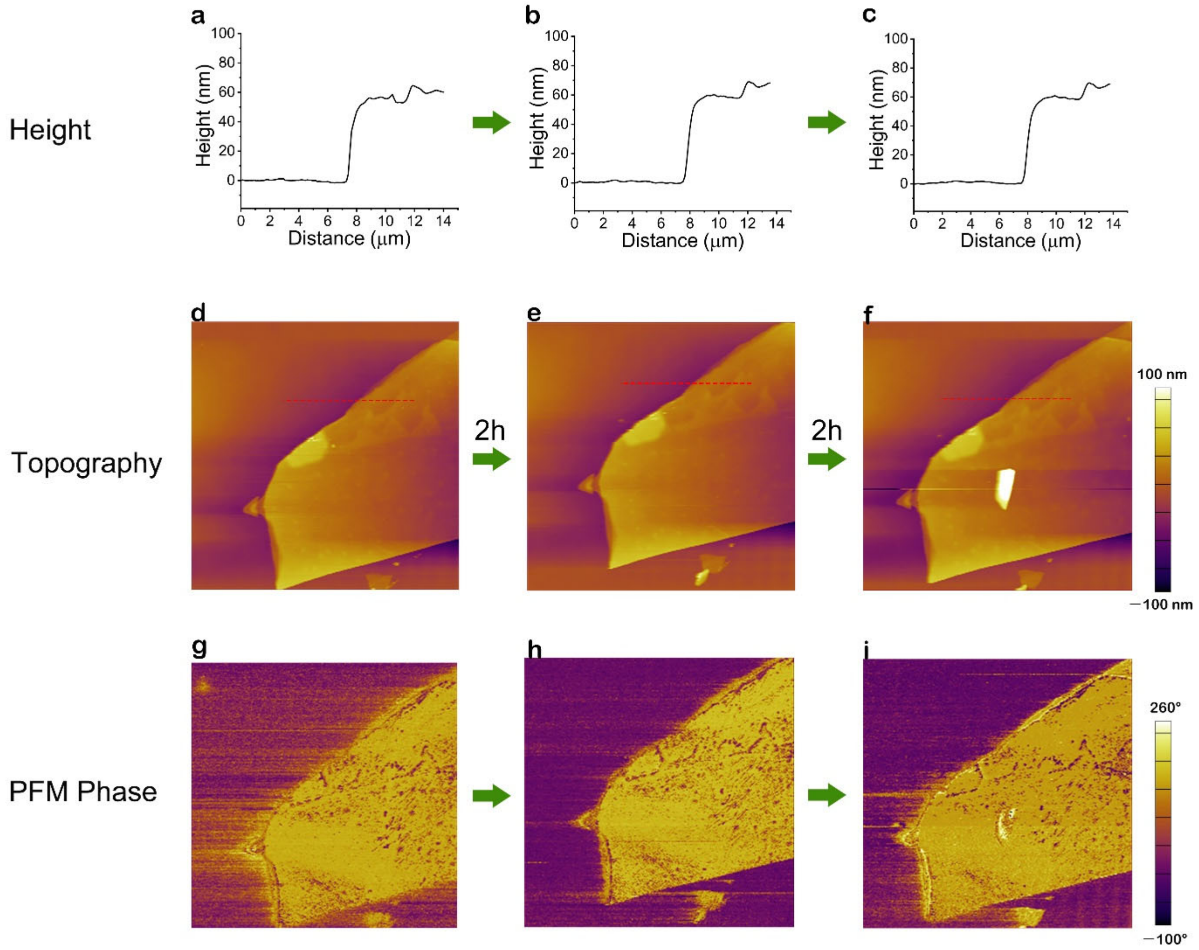


**Supplementary Figure 7. Polarization states of CIPS after acidic aqueous solution treatment.** Time evolution of the height (**a-c**), topography (**d-f**) and corresponding phase (**g-h**) of CIPS after exposure to $H_2SO_4$ aqueous solution (pH = 3).

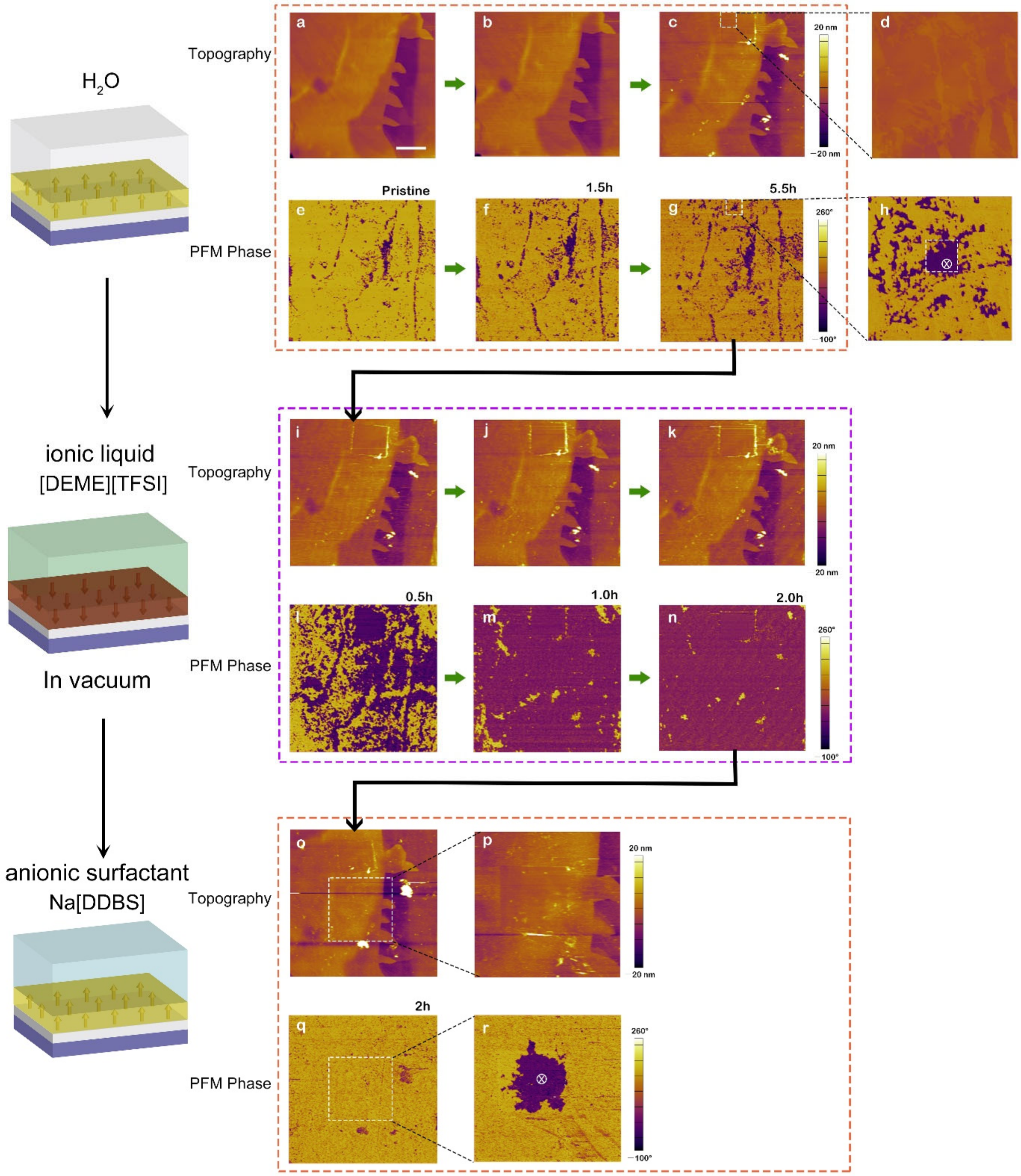


**Supplementary Figure 8. Continuous experiments on the same sample using $H_2O$, [DEME][TFSI] and Na[DDBS], respectively.** The topography (**a-c**, **i-k**, **o**) exhibits no change, while the corresponding PFM phase changes significantly, i.e. the polarization cannot be switched obviously by $H_2O$ (**e-g**), but can be reversed by ionic liquid [DEME][TFSI] (**l-n**) and reversed back by Na[DDBS] solution (**q**). The direction of polarization is distinguished by electrical switching (**d** and **h**; **p** and **r**). Scale bar in (**a**) is 3 μm.

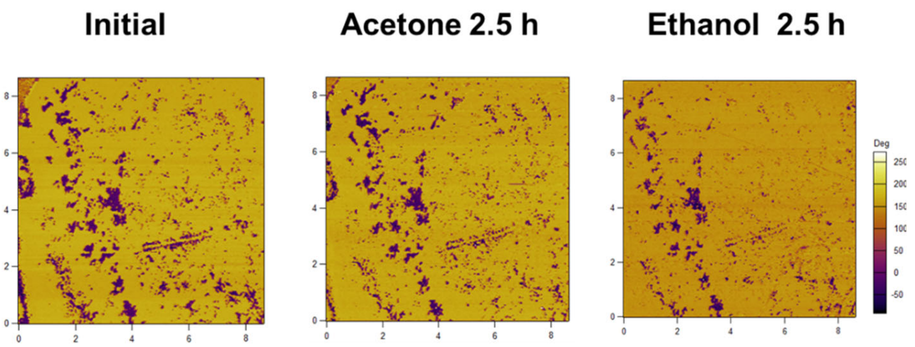


**Supplementary Figure 9. Domain structures before and after the treatment by acetone and ethanol, respectively.** Length unit is μm.

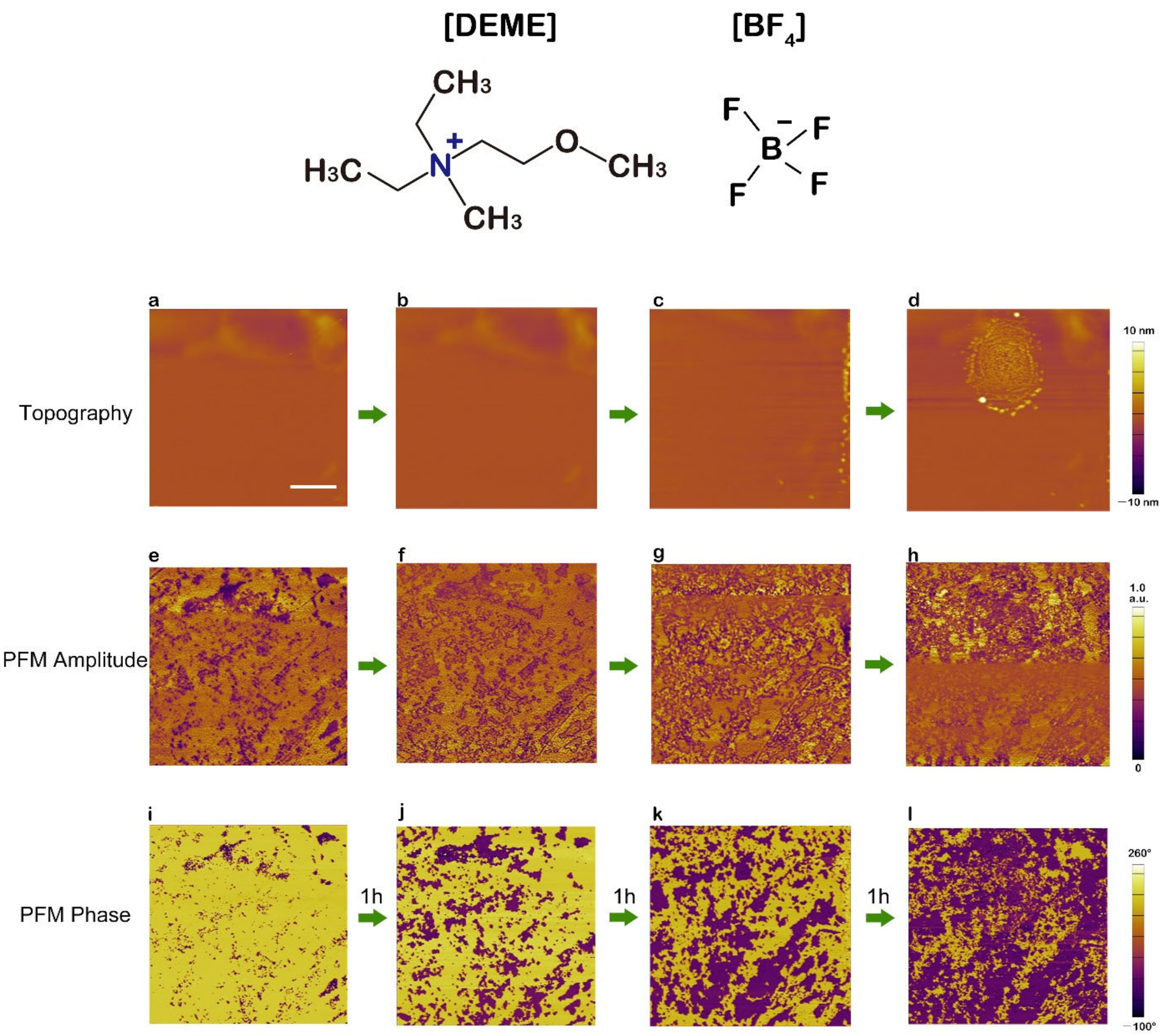


**Supplementary Figure 10. Ionic liquid [DEME][$BF_4$]-induced polarization switching of CIPS.** The molecular structure of [DEME][$BF_4$] (up panel). Time evolution of the topography (**a-d**), PFM amplitude signals (**e-h**) and corresponding phase images (**i-l**) of a CIPS flake after exposure to [DEME][$BF_4$]. Scale bar in (**d**) is 2.5 μm.

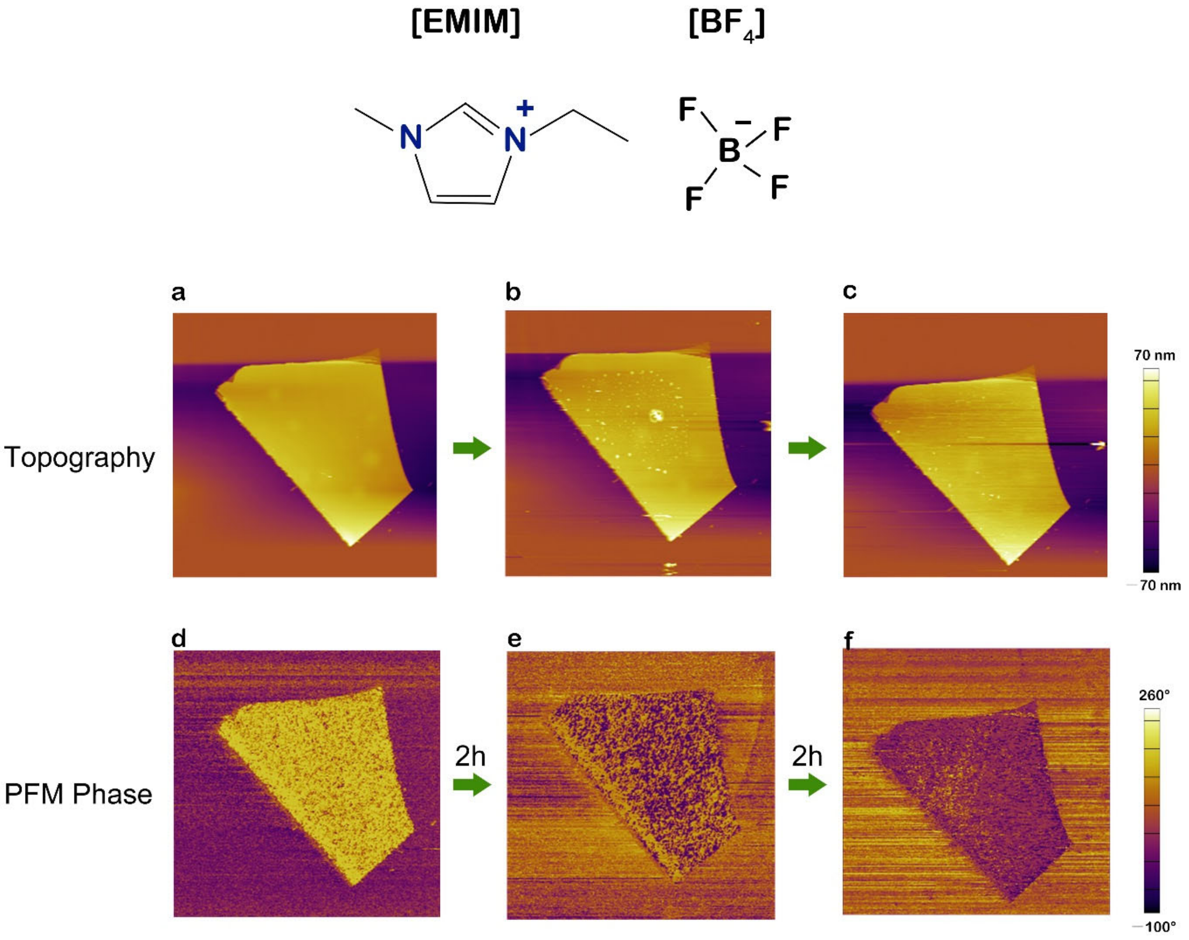


**Supplementary Figure 11. Ionic liquid [EMIM][$BF_4$]-induced polarization switching of CIPS.** The molecular structure of [EMIM][$BF_4$] (up panel). Time evolution of the topography (**a**-**c**) and corresponding phase images (**d**-**f**) of a CIPS flake after exposure to [EMIM][$BF_4$].

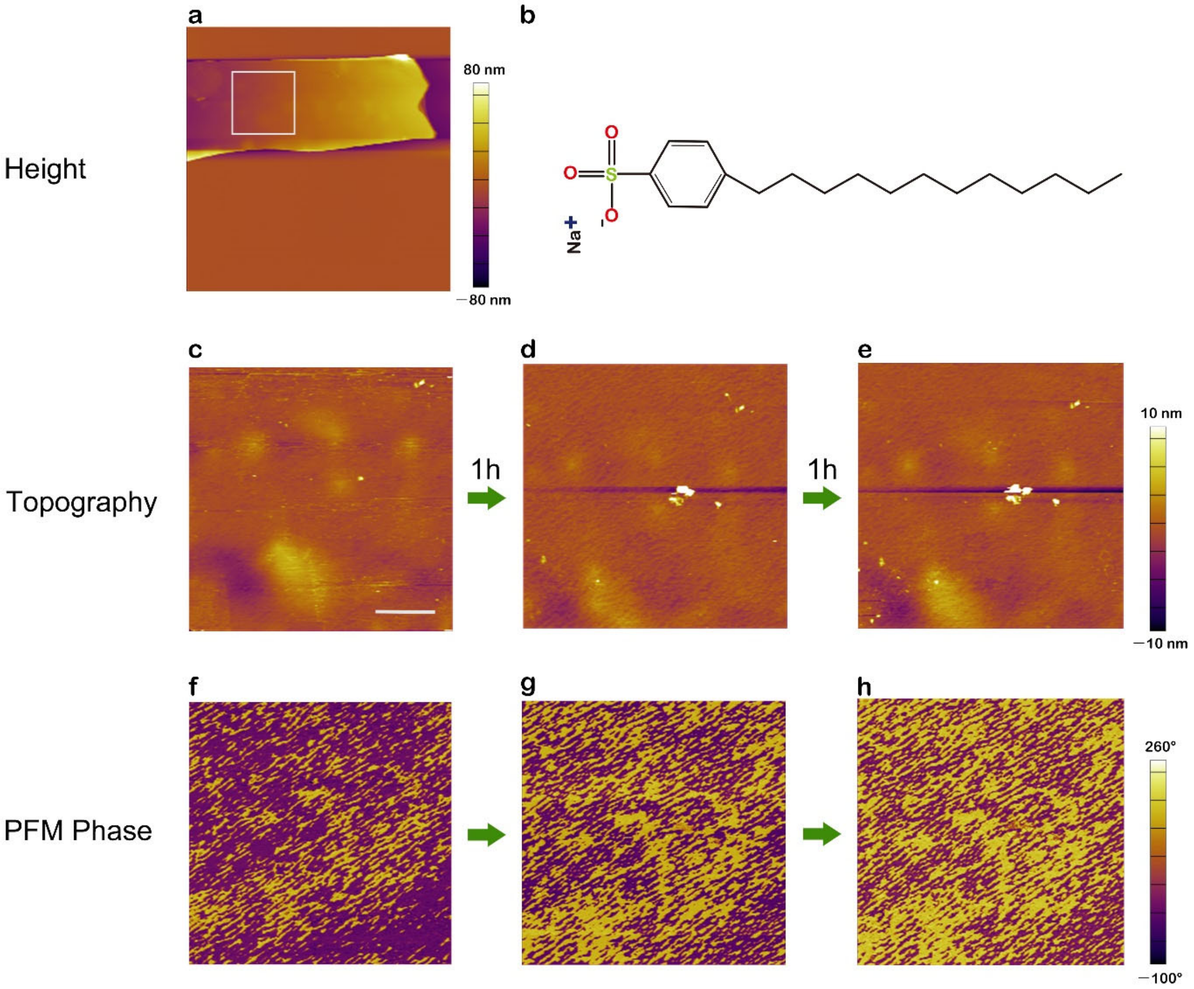


**Supplementary Figure 12. Na[DDBS] solution-induced polarization switching of CIPS. a** Topography of the CIPS flake used for PFM measurements in (**c**-**h**). **b** The molecular structure of Na[DDBS]. Time evolution of the topography (**c**-**e**) and corresponding phase images (**f**-**h**) of the CIPS flake in (**a**) after exposure to Na[DDBS] solution. Scale bar in (**c**) is 2 µm.

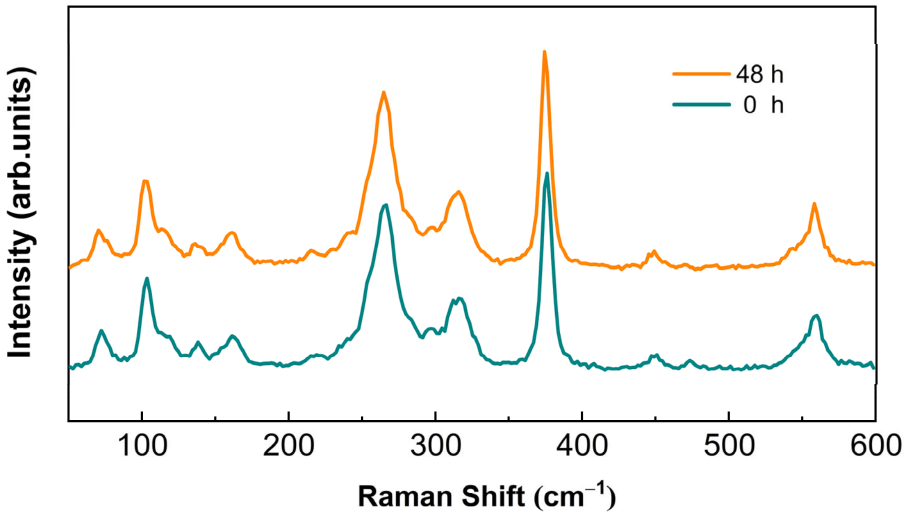


**Supplementary Figure 13. Comparison of Raman spectra before and after Na[DDBS] solution treatment.**

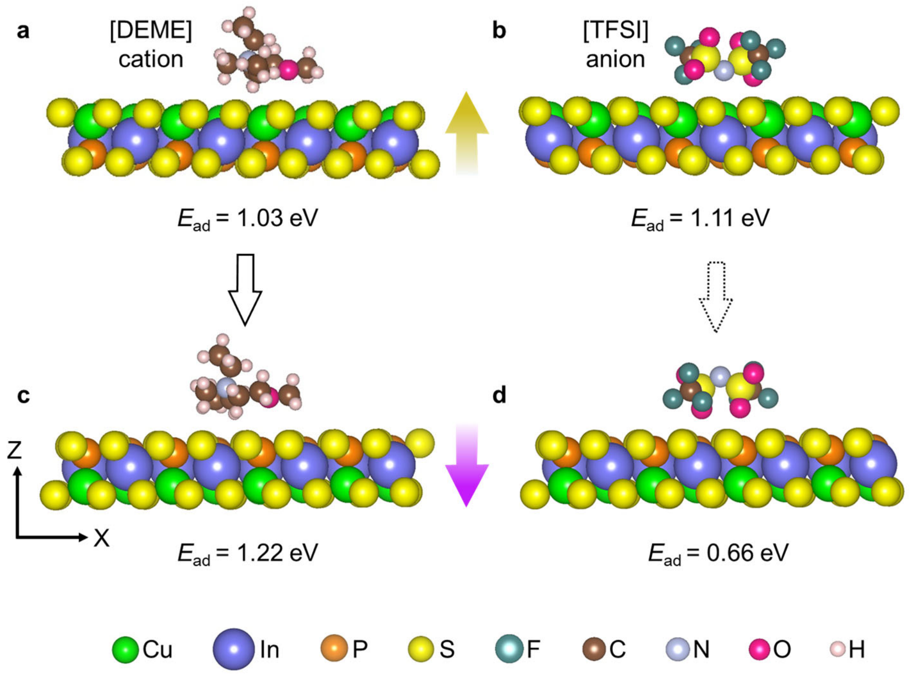


**Supplementary Figure 14. Comparison of adsorption energies of [DEME] cations and [TFSI] anions onto the CIPS surface.** The yellow and purple arrows represent upward (**a, b**) and downward polarization (**c, d**), respectively.

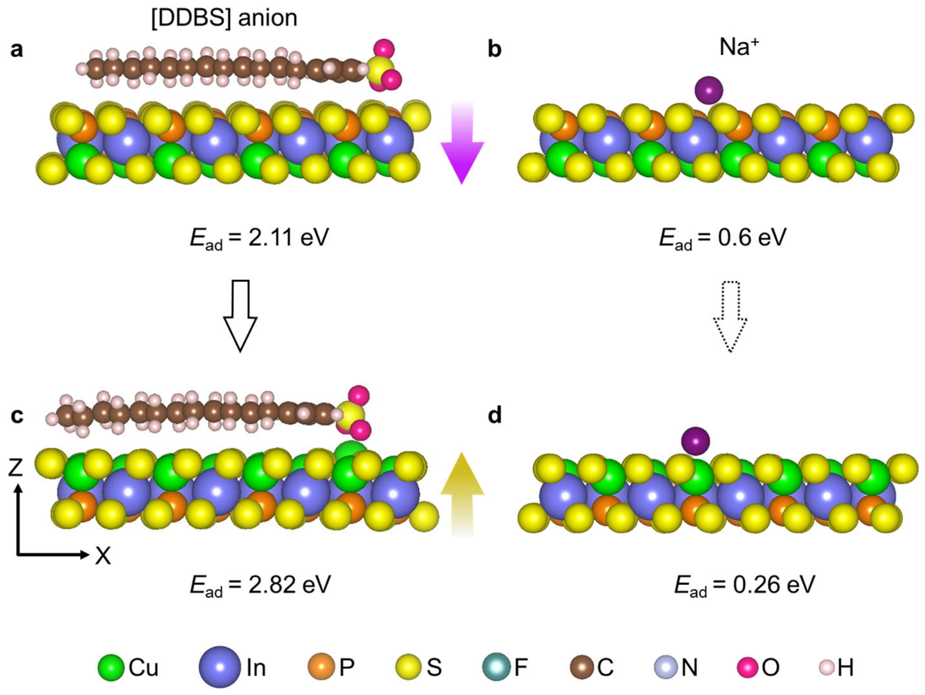


**Supplementary Figure 15. Comparison of adsorption energies of [DDBS] anions and $Na^+$ onto the CIPS surface.** The yellow and purple arrows represent upward (**c, d**) and downward polarization (**a, b**), respectively.

**Supplementary Note 2.**

Supplementary Figs. 14 and 15 shows the comparison of adsorption energies of four adsorption scenarios. The adsorption energy can be expressed as: $E_{ad} = E_{surface} + E_{ion} - E_{surface\text{-}ion}$ where $E_{surface}$, $E_{ion}$ and $E_{surface\text{-}ion}$ is the total energy of bare CIPS surface, an isolated ion, and the optimized CIPS with adsorbed species, respectively. As shown in Supplementary Fig. 14c, it is the most stable state that [DEME] cations adsorb onto the downward polarized surface, and the most unstable state is the case that [TFSI] anions adsorb onto the downward polarized surface (Supplementary Fig. 14d). Now considering the CIPS surface with initial upward polarization (Supplementary Fig. 14a, b), [DEME] cations and [TFSI] anions have almost the same adsorption energy ($\Delta E_{ad}$ is only 0.08 eV). That is, [DEME] cations and [TFSI] anions have the same probability to adsorb onto the surface. When the adsorption of [DEME] cations occurs (Supplementary Fig. 14a), the polarization would be switched leading to the most stable state Supplementary Fig. 14c). In contrast, the adsorption of [TFSI] anions (Supplementary Fig. 14b) would not induce the polarization switching, and even if the polarization was switched, it would result in the most unstable state (Supplementary Fig. 14d). Therefore, it is to be expected that downward domains with the most stable state will be expanded with the increase of the adsorption quantity of [DEME] cations (Fig. 4g in the main text), which is consistent with the observed dynamic switching process in the manuscript (Figure 1d). These results not only confirm the role of [DEME] cations in the polarization switching process, but also explain why anions cannot prevent the switching.

As for the CIPS-Na[DDBS] interface, the adsorption energy of [DDBS] anion is always much larger than that of $Na^+$ both on downward and upward polarized surfaces revealing high adsorbability of [DDBS] on the CIPS surface (Supplementary Fig. 15). Therefore, the adsorption of [DDBS] anions can induce polarization switching via the same mechanism.

If considering the partial screening of CIPS surfaces in air, the positive bound charges on the surface (upward polarized domains) can be screened by compensating charges. The electrostatic repulsion between [DEME] cations and positive bound charges should be ignored leading to an

increase in the adsorption energy of [DEME] cations (Supplementary Fig. 14a). Similarly, the electrostatic attraction between [TFSI] anions and positive bound charges also should be screened by compensating charges resulting in a decrease in the adsorption energy of [TFSI] anions (Supplementary Fig. 14b). As a result, the adsorption of [DEME] cations will be energetically preferred compared to [TFSI] anions. Therefore, surface screening is favorable for the adsorption of [DEME] cations and suppress the adsorption of [TFSI] anions.

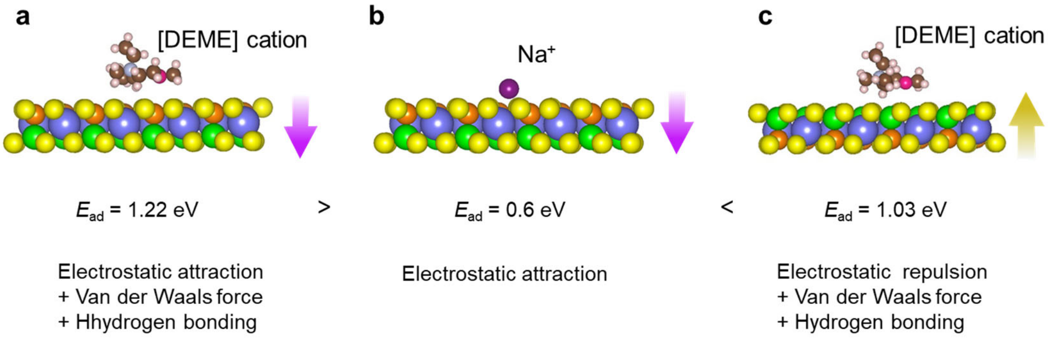


**Supplementary Figure 16. Comparison of adsorption energies of large [DEME] cation and small $Na^+$ ion at CIPS surfaces.** The purple and yellow arrows represent downward (**a**, **b**) and upward polarization (**c**), respectively.

**Supplementary Note 3.**

To clarify the relative strength of surface adsorbability quantitatively compared to electrostatic interaction, the comparison of adsorption energies of small $Na^+$ ion and large [DEME] cation at CIPS surfaces has been made. The adsorption of small $Na^+$ ions can be seen as the result of purely electrostatic interaction, but for [DEME] cations, the adsorption is driven by the combined effect of electrostatic interaction, van der Waals interaction and hydrogen bonding with CIPS surface. The adsorption energy can be expressed as: $E_{ad} = E_{surface} + E_{ion} - E_{surface\text{-}ion}$ where $E_{surface}$, $E_{ion}$ and $E_{surface\text{-}ion}$ is the total energy of bare CIPS surface, an isolated ion, and the optimized CIPS with adsorbed species, respectively. As shown in Figure R4, the adsorption energy of cation [DEME] (1.22 eV, Figure 16a) is much larger than that of $Na^+$ (0.6 eV, Figure 16b) even though they have the same amount of charge (1 unit charge) and adsorb onto the same surface (downward polarized surface). This suggests that van der Waals interactions and hydrogen bonding has a strong effect on the surface adsorption of [DEME] cations. More impressively, considering the case that cations [DEME] adsorb onto the upward polarized surface, the adsorption energy (1.03 eV, Figure 16c) is still much larger than that of $Na^+$ although electrostatic repulsion makes against the ion adsorption of [DEME] cation intuitively. This comparison clearly proves the fact that van der Waals interactions and hydrogen bonding play a crucial role in the ion adsorption process, and can be strong enough to overcome Coulomb interactions.

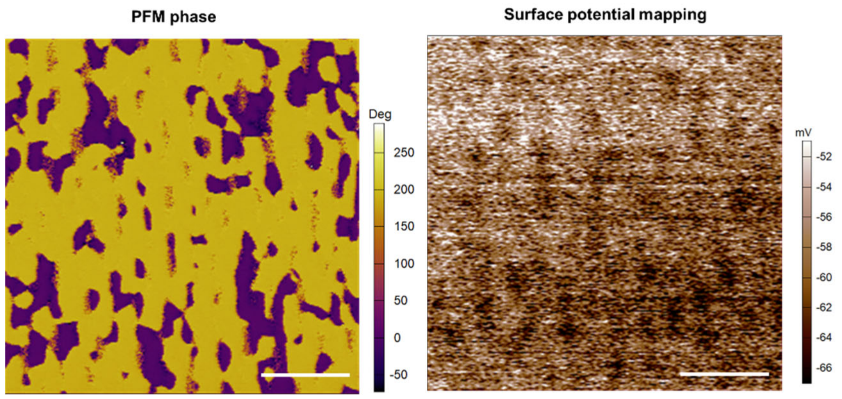


**Supplementary Figure 17. Initial domain structures of a fresh nanoflake and corresponding surface potential.** Scale bar is 500 nm. As shown in Supplementary Fig. 17, there is no significant surface potential difference between upward and downward domains in a fresh flake. This result suggests the surface screening effect of spontaneous polarization (bond charges) is partially realized by charges adsorbed from the ambient. Note that there is possibility that the small potential difference cannot be detected by our KPFM equipment due to the relatively small ferroelectric polarization (~3.2 $\mu C/cm^2$).
